# Comparison of the Complexity of Patients Seen by Different Medical Subspecialists in a Universal Health Care System

**DOI:** 10.1001/jamanetworkopen.2018.4852

**Published:** 2018-11-30

**Authors:** Marcello Tonelli, Natasha Wiebe, Braden J. Manns, Scott W. Klarenbach, Matthew T. James, Pietro Ravani, Neesh Pannu, Jonathan Himmelfarb, Brenda R. Hemmelgarn

**Affiliations:** 1 Department of Medicine, University of Calgary, Calgary, Alberta, Canada; 2 Department of Medicine, University of Alberta, Edmonton, Alberta, Canada; 3 Department of Medicine, University of Washington, Seattle

## Abstract

**Importance:**

Clinical experience suggests that there are substantial differences in patient complexity across medical specialties, but empirical data are lacking.

**Objective:**

To compare the complexity of patients seen by different types of physician in a universal health care system.

**Design, Setting, and Participants:**

Population-based retrospective cohort study of 2 597 127 residents of the Canadian province of Alberta aged 18 years and older with at least 1 physician visit between April 1, 2014 and March 31, 2015. Data were analyzed in September 2018.

**Exposures:**

Type of physician seeing each patient (family physician, general internist, or 11 types of medical subspecialist) assessed as non–mutually exclusive categories.

**Main Outcomes and Measures:**

Nine markers of patient complexity (number of comorbidities, presence of mental illness, number of types of physicians involved in each patient’s care, number of physicians involved in each patient’s care, number of prescribed medications, number of emergency department visits, rate of death, rate of hospitalization, rate of placement in a long-term care facility).

**Results:**

Among the 2 597 127 participants, the median (interquartile range) age was 46 (32-59) years and 54.1% were female. Over 1 year of follow-up, 21 792 patients (0.8%) died, the median (range) number of days spent in the hospital was 0 (0-365), 8.1% of patients had at least 1 hospitalization, and the median (interquartile range) number of prescribed medications was 3 (1-7). When the complexity markers were considered individually, patients seen by nephrologists had the highest mean number of comorbidities (4.2; 95% CI, 4.2-4.3 vs [lowest] 1.1; 95% CI, 1.0-1.1), highest mean number of prescribed medications (14.2; 95% CI, 14.2-14.3 vs [lowest] 4.9; 95% CI, 4.9-4.9), highest rate of death (6.6%; 95% CI, 6.3%-6.9% vs [lowest] 0.1%; 95% CI, <0.1%-0.2%), and highest rate of placement in a long-term care facility (2.0%; 95% CI, 1.8%-2.2% vs [lowest] <0.1%; 95% CI, <0.1%-0.1%). Patients seen by infectious disease specialists had the highest complexity as assessed by the other 5 markers: rate of a mental health condition (29%; 95% CI, 28%-29% vs [lowest] 14%; 95% CI, 14%-14%), mean number of physician types (5.5; 95% CI, 5.5-5.6 vs [lowest] 2.1; 95% CI, 2.1-2.1), mean number of physicians (13.0; 95% CI, 12.9-13.1 vs [lowest] 3.8; 95% CI, 3.8-3.8), mean days in hospital (15.0; 95% CI, 14.9-15.0 vs [lowest] 0.4; 95% CI, 0.4-0.4), and mean emergency department visits (2.6; 95% CI, 2.6-2.6 vs [lowest] 0.5; 95% CI, 0.5-0.5). When types of physician were ranked according to patient complexity across all 9 markers, the order from most to least complex was nephrologist, infectious disease specialist, neurologist, respirologist, hematologist, rheumatologist, gastroenterologist, cardiologist, general internist, endocrinologist, allergist/immunologist, dermatologist, and family physician.

**Conclusion and Relevance:**

Substantial differences were found in 9 different markers of patient complexity across different types of physician, including medical subspecialists, general internists, and family physicians. These findings have implications for medical education and health policy.

## Key Points

**Question:**

Are there differences in the complexity of patients seen by different types of physicians?

**Findings:**

In this population-based cohort study of 2.5 million Canadian adults, there were substantial differences in markers of complexity for patients seen by different types of physicians, including medical subspecialists. Patients seen by nephrologists, infectious disease specialists, and neurologists were consistently more complex, whereas patients seen by allergists, dermatologists, and family physicians consistently tended to be less complex.

**Meaning:**

Substantial between-specialty differences were found in 9 different markers of patient complexity. The relative rank of the different specialties studied is less important than the finding that there are wide variations in complexity between specialties, which has implications for medical education and health policy.

## Teaser

This cohort study compares 9 markers of patient complexity among patients treated by physicians in different specialties in Alberta, Canada, using data collected by the province’s universal health care system.

## Introduction

Patient complexity can be defined as an interaction between the “personal, social, and clinical aspects of the patient’s experience”^1^ that complicates patient care. For example, increasing age and comorbidity, social factors (eg, poverty and lower level of education), treatment characteristics (eg, number of medications), and contextual factors (eg, residence in long-term care) all influence perceived patient complexity^2^—and the prevalence of complexity appears to be increasing in health systems worldwide. There is general agreement that patient complexity increases the time and resources required to provide optimal care. However, payments to health care facilities and physicians are both frequently based on patient volume rather than patient complexity.^3,4,5^ Even in systems that are not fee-for-service based, the time allotted to see a given number of patients often does not account for patient complexity.^6^

Clinical experience suggests that the complexity of patients varies substantially between different medical specialties, although empirical data are lacking. To better understand the complexity of patients receiving care from different types of physicians, enabling a better estimation of the likely resource needs of these clinical populations, we compared the complexity of patients seen by different types of physician in a universal health care system. Since there is no consensus of how complexity should be measured,^7^ we used the number of comorbidities, the presence of mental illness, the number of types of physicians involved in each patient’s care, the number of physicians involved in each patient’s care, the number of prescribed medications, the number of emergency department visits, and the rate of adverse clinical outcomes (death, all-cause hospitalization, and placement in a long-term care facility) as proxies for complexity. We hypothesized that we would observe substantial differences in these measures of complexity across patients seen by the different types of physician in our study.

## Methods

This retrospective population-based cohort study is reported according to the Strengthening the Reporting of Observational Studies in Epidemiology (STROBE) reporting guideline.^8^ The institutional review boards at the University of Alberta and the University of Calgary approved this study and waived the requirement for participants to provide consent.

### Data Sources and Cohort

We used a previously described database^9,10,11^ that incorporates data from Alberta Health (the provincial health ministry), including physician claims, hospitalizations, ambulatory care utilization, and Alberta pharmaceutical network data; the database also collects information from the clinical laboratories in Alberta, Canada. This database has population-based coverage of a geographically defined area, including demographic characteristics, health services utilization, and clinical outcomes. Indigenous status includes people who are registered as First Nations or recognized as Inuit. Additional information on the database is available elsewhere, including the validation of selected data elements and the standardization and calibration of serum creatinine assays.^12^ All individuals registered with Alberta Health were included in the database (all Alberta residents are eligible for insurance coverage by Alberta Health and >99% participate in coverage). The database was used to assemble a cohort of adults (aged ≥18 years) who resided in Alberta on April 1, 2014. Patients’ residential postal codes were used to classify them as residing in a rural area^13^ or in a lower-income neighborhood using the Statistics Canada definition of lowest neighborhood income quintile.^13^ We followed patients from April 1, 2014 (baseline), until death, out-migration from Alberta, or study end (March 31, 2015), whichever was earliest.

### Comorbidities

Comorbidities were defined using a previously published framework with 29 validated algorithms as applied to Canadian physician claims data, each of which had positive predictive values of 70% or greater as compared with a gold-standard measure such as medical record review.^14^ These comorbidities were alcohol misuse, asthma, atrial fibrillation, lymphoma, nonmetastatic cancer (breast, cervical, colorectal, pulmonary, and prostate cancer), metastatic cancer, chronic heart failure, chronic pain, chronic obstructive pulmonary disease, chronic hepatitis B, cirrhosis, severe constipation, dementia, depression, diabetes, epilepsy, hypertension, hypothyroidism, inflammatory bowel disease, irritable bowel syndrome, multiple sclerosis, myocardial infarction, Parkinson disease, peptic ulcer disease, peripheral vascular disease, psoriasis, rheumatoid arthritis, schizophrenia, and stroke or transient ischemic attack. Each patient was classified with respect to the presence or absence of these 29 chronic conditions at baseline.^15^ Detailed methods for classifying comorbidity status and the specific algorithms used are found elsewhere.^14^ The presence of chronic kidney disease was also ascertained, captured using the single closest outpatient measurement of creatinine and albuminuria within 1 year of baseline, and defined based on international guidelines.^15^

### Physician Care

We used outpatient and inpatient physician claims data to determine the physician or physicians who saw each patient. A single claim from a given physician for an individual patient in the year prior to baseline was sufficient to define the former as being seen by the latter. We focused on physicians whose practices are nonsurgical, including family physicians, general internists, and medical subspecialists. Medical subspecialists were defined as physicians with qualifications in cardiology, clinical immunology and allergy, dermatology, endocrinology, gastroenterology, hematology, infectious diseases, nephrology, neurology, rheumatology, or respiratory medicine. In all analyses, we excluded patients who did not receive care from any of these physicians in the year prior to baseline. Medical oncologists and specialists in geriatric medicine were excluded because in Alberta, these physicians are predominantly paid by salary and do not submit claims for most of their clinical encounters. Groups were not mutually exclusive, meaning that a patient who was seen by a family physician, a cardiologist, and a clinical allergist/immunologist would be classified as being seen by all of these physicians.

### Markers of Complexity

We considered 9 markers as proxies for patient complexity. Seven were measured in the year prior to follow-up to minimize the impact of the competing risk of mortality on nondeath outcomes: the number of comorbidities, the number of uniquely prescribed medications (defined by unique chemical entities as assessed by prescriptions filled), the presence of a mental health condition (defined by alcohol misuse, depression, or schizophrenia), the number of physician types seen by each patient, the total number of physicians involved in each patient’s care, the number of days spent in a hospital, and the number of emergency department visits. The remaining 2 markers, the risk of new placement into long-term care and the risk of all-cause death, were measured during the year of follow-up.

For analyses using physician type as an outcome, we considered the medical subspecialties listed in the Physician Care section, general internists and family physicians, and all other physicians who submit claims for patient visits and procedures. Nonphysician health professionals, such as chiropractors, dentists, and dieticians, were not included.

### Statistical Analysis

We did analyses with Stata MP statistical software version 15.0 (StataCorp) and reported baseline descriptive statistics as counts and percentages. Probabilities and means were reported where appropriate. Confidence intervals for probabilities and means were calculated using exact binomial and exact Poisson methods. We used unadjusted logistic regression to determine the associations between scenarios of physician care and the ratio of odds for dichotomous outcomes and unadjusted Poisson regression to determine the associations between scenarios of physician care and the ratio of means for count outcomes. Between-group variability (physician groups) was measured using χ^2^ tests of equality between model coefficient estimates. The threshold for statistical significance was set at 2-sided *P* < .05. Because the emphasis of this article was to capture the actual complexity of patients seen by the different physician types (rather than to examine the factors responsible for any observed differences, or to test for an independent association of complexity with physician group), we did not do adjusted analyses. Using results from the regressions, the specialties were uniformly ranked for each complexity marker, with the highest ratio (rate ratio or odds ratio) receiving the highest rank. The ranks were then summed across the 9 complexity markers giving an overall complexity rank for each physician type. In sensitivity analyses, we considered the patient-visit (1 claim) as the unit of analysis rather than a patient, meaning that patients who were seen more frequently were given more weight. In further sensitivity analyses, we required at least 2 claims (on ≥2 days), or at least 3 claims (on ≥3 days) to be sufficient for a given physician to have seen an individual patient (in the year prior to baseline). We also considered the 1-year cohort beginning in April 1, 2009.

## Results

### Characteristics of Study Patients

Patient flow is shown in eFigure 1 in the Supplement. Overall 1 039 403 patients (28.6%) were excluded because they were not seen by at least 1 family physician, general internist, or medical subspecialist during the study period, leaving 2 597 127 patients in the cohort. No data were missing except for rural status (0.5%) and lowest neighborhood income quintile (5.6%).

The median (interquartile range) age of the participants was 46 (32-59) years and 54.1% were female. The median (interquartile range) number of comorbidities for all patients was 1 (0-2); 833 223 patients (32.1%) had more than 1 comorbidity; 476 079 (18.3%) had 3 or more comorbidities, and 146 993 (5.7%) had 5 or more comorbidities. Over 1 year of follow-up, 21 792 (0.8%) died, the median (range) days spent in the hospital was 0 (0-365) (211 384 [8.1%] with ≥1 hospitalization), and the median (interquartile range) number of prescribed medications was 3 (1-7). Baseline characteristics of the patients by physician group are shown in Table 1. Some specialties were more likely than others to see patients with characteristics that might contribute to complexity. For example, a greater proportion of older patients were seen by cardiologists, hematologists, and nephrologists. Patients of indigenous origin were most often seen by nephrologists, neurologists, and rheumatologists. Patients on social assistance were more often seen by infectious disease specialists, nephrologists, and neurologists. Patients residing in rural communities were more likely to see family physicians, nephrologists, and rheumatologists.

**Table 1.** Demographics and Clinical Characteristics by Physician Type

Characteristic	No. (%)												
	Nephrologist	Infectious Disease Specialist	Neurologist	Respirologist	Hematologist	Rheumatologist	Gastroenterologist	Cardiologist	General Internist	Endocrinologist	Allergist/Immunologist	Dermatologist	Family Physician
No.	26 243	17 257	72 693	67 159	11 797	12 876	90 903	180 135	462 194	11 339	11 096	177 497	2569 697
Age, y													
18-59	10 616 (40.5)	11 669 (67.6)	45 797 (63.0)	36 624 (54.5)	6009 (50.9)	7703 (59.8)	53 434 (58.8)	88 878 (49.3)	268 479 (58.1)	7336 (64.7)	9806 (88.4)	114 051 (64.3)	193 3445 (75.2)
60-79	10 967 (41.8)	4306 (25.0)	20 892 (28.7)	24 512 (36.5)	4407 (37.4)	4494 (34.9)	31 765 (34.9)	71 118 (39.5)	154 514 (33.4)	3416 (30.1)	1202 (10.8)	49 988 (28.2)	515 426 (20.1)
≥80	4660 (17.8)	1282 (7.4)	6004 (8.3)	6023 (9.0)	1381 (11.7)	679 (5.3)	5704 (6.3)	20 139 (11.2)	39 201 (8.5)	587 (5.2)	88 (0.8)	13 458 (7.6)	120 826 (4.7)
Female	11 945 (45.5)	8001 (46.4)	41 909 (57.7)	34 940 (52.0)	5947 (50.4)	8799 (68.3)	49 476 (54.4)	83 136 (46.2)	239 213 (51.8)	7877 (69.5)	7252 (65.4)	107 327 (60.5)	139 3471 (54.2)
Indigenous	897 (3.4)	993 (5.8)	1670 (2.3)	1509 (2.2)	170 (1.4)	525 (4.1)	1812 (2.0)	3094 (1.7)	10 103 (2.2)	321 (2.8)	159 (1.4)	1972 (1.1)	79 013 (3.1)
Social assistance	2125 (8.1)	1937 (11.3)	603 (9.0)	430 (6.0)	610 (5.2)	591 (4.6)	357 (4.4)	652 (3.6)	2032 (4.4)	501 (4.4)	197 (1.8)	3741 (2.1)	80 273 (3.1)
Lowest neighborhood income quintile	5716 (22.7)	4397 (26.9)	13 714 (19.8)	13 422 (20.9)	1941 (17.3)	2217 (18.1)	14 692 (17.0)	32 587 (19.0)	88 758 (20.3)	2058 (19.2)	1484 (14.2)	24 697 (14.6)	472 360 (19.5)
Rural	2428 (9.3)	989 (5.8)	6095 (8.4)	5641 (8.4)	774 (6.6)	1454 (11.3)	5379 (5.9)	11 016 (6.1)	27 781 (6.0)	720 (6.4)	686 (6.2)	8771 (5.0)	251 342 (9.8)
Morbidity	1858 (7.1)	1914 (11.1)	3781 (5.2)	3240 (4.8)	503 (4.3)	376 (2.9)	4893 (5.4)	6174 (3.4)	18 285 (4.0)	401 (3.5)	155 (1.4)	3377 (1.9)	74 544 (2.9)
Alcohol misuse	1658 (6.3)	1099 (6.4)	3540 (4.9)	7579 (11.3)	589 (5.0)	624 (4.8)	3829 (4.2)	6710 (3.7)	17 766 (3.8)	424 (3.7)	617 (5.6)	4850 (2.7)	61 756 (2.4)
Asthma	3780 (14.4)	1447 (8.4)	4528 (6.2)	5895 (8.8)	1150 (9.7)	594 (4.6)	5087 (5.6)	24 594 (13.7)	30 281 (6.6)	552 (4.9)	119 (1.1)	7184 (4.0)	69 741 (2.7)
Atrial fibrillation	592 (2.3)	322 (1.9)	481 (0.7)	714 (1.1)	2372 (20.1)	145 (1.1)	755 (0.8)	1377 (0.8)	3787 (0.8)	109 (1.0)	26 (0.2)	1177 (0.7)	7616 (0.3)
Lymphoma	663 (2.5)	434 (2.5)	833 (1.1)	1522 (2.3)	508 (4.3)	131 (1.0)	1809 (2.0)	2129 (1.2)	6355 (1.4)	331 (2.9)	35 (0.3)	1632 (0.9)	15 079 (0.6)
Metastatic cancer	1538 (5.9)	806 (4.7)	2572 (3.5)	4364 (6.5)	731 (6.2)	437 (3.4)	5003 (5.5)	7764 (4.3)	18 848 (4.1)	371 (3.3)	158 (1.4)	6501 (3.7)	59 029 (2.3)
Single-site cancer	6139 (23.4)	1995 (11.6)	4520 (6.2)	7859 (11.7)	1310 (11.1)	691 (5.4)	5312 (5.8)	25 445 (14.1)	30 808 (6.7)	650 (5.7)	92 (0.8)	6055 (3.4)	68 283 (2.7)
Chronic heart failure	22 501 (85.7)	6309 (36.6)	22 129 (30.4)	22 038 (32.8)	4690 (39.8)	4480 (34.8)	26 936 (29.6)	62 627 (34.8)	146 468 (31.7)	4051 (35.7)	1667 (15.0)	41 895 (23.6)	489 323 (19.0)
Chronic kidney disease	5513 (21.0)	3831 (22.2)	19 737 (27.2)	13 957 (20.8)	2370 (20.1)	7097 (55.1)	17 116 (18.8)	31 181 (17.3)	81 563 (17.6)	1973 (17.4)	1485 (13.4)	26 084 (14.7)	301 447 (11.7)
Chronic pain	6669 (25.4)	3469 (20.1)	10 563 (14.5)	25 724 (38.3)	2111 (17.9)	1982 (15.4)	13 075 (14.4)	30 161 (16.7)	67 603 (14.6)	1252 (11.0)	825 (7.4)	16 685 (9.4)	216 422 (8.4)
Chronic pulmonary	128 (0.5)	415 (2.4)	121 (0.2)	151 (0.2)	46 (0.4)	23 (0.2)	1003 (1.1)	473 (0.3)	1723 (0.4)	54 (0.5)	9 (0.1)	308 (0.2)	4421 (0.2)
Viral hepatitis B	427 (1.6)	357 (2.1)	299 (0.4)	433 (0.6)	197 (1.7)	45 (0.3)	2397 (2.6)	746 (0.4)	2445 (0.5)	230 (2.0)	8 (0.1)	395 (0.2)	4271 (0.2)
Cirrhosis	1479 (5.6)	761 (4.4)	2742 (3.8)	2251 (3.4)	482 (4.1)	324 (2.5)	4747 (5.2)	4432 (2.5)	11 599 (2.5)	288 (2.5)	151 (1.4)	2926 (1.6)	33 810 (1.3)
Severe constipation	1746 (6.7)	811 (4.7)	3813 (5.2)	2014 (3.0)	407 (3.5)	187 (1.5)	2154 (2.4)	4676 (2.6)	13 016 (2.8)	197 (1.7)	33 (0.3)	2617 (1.5)	39 900 (1.6)
Dementia	4514 (17.2)	3793 (22.0)	16 954 (23.3)	12 114 (18.0)	1994 (16.9)	2122 (16.5)	15 346 (16.9)	24 379 (13.5)	69 619 (15.1)	1769 (15.6)	1492 (13.4)	22 304 (12.6)	302 058 (11.8)
Depression	11 106 (42.3)	3661 (21.2)	11 369 (15.6)	12 439 (18.5)	2146 (18.2)	1695 (13.2)	14 267 (15.7)	37 485 (20.8)	91 834 (19.9)	4055 (35.8)	546 (4.9)	17 745 (10.0)	256 469 (10.0)
Diabetes	1055 (4.0)	736 (4.3)	11 800 (16.2)	1798 (2.7)	407 (3.5)	392 (3.0)	2409 (2.7)	4307 (2.4)	10 845 (2.3)	310 (2.7)	160 (1.4)	3131 (1.8)	43 934 (1.7)
Epilepsy	20 726 (79.0)	6786 (39.3)	29 139 (40.1)	30 890 (46.0)	5443 (46.1)	5178 (40.2)	36 691 (40.4)	99 500 (55.2)	210 869 (45.6)	4445 (39.2)	1774 (16.0)	55 197 (31.1)	670 924 (26.1)
Hypertension	4528 (17.3)	1787 (10.4)	9712 (13.4)	967 (13.8)	1709 (14.5)	1886 (14.6)	11 570 (12.7)	23 971 (13.3)	64 777 (14.0)	2932 (25.9)	1044 (9.4)	20 008 (11.3)	228 621 (8.9)
Hypothyroidism	662 (2.5)	455 (2.6)	1285 (1.8)	1176 (1.8)	323 (2.7)	437 (3.4)	11 223 (12.3)	2468 (1.4)	8673 (1.9)	234 (2.1)	152 (1.4)	3029 (1.7)	28 269 (1.1)
Inflammatory bowel disease	798 (3.0)	503 (2.9)	3002 (4.1)	2300 (3.4)	443 (3.8)	478 (3.7)	6495 (7.1)	4970 (2.8)	14 249 (3.1)	380 (3.4)	420 (3.8)	5544 (3.1)	50 022 (1.9)
Irritable bowel syndrome	363 (1.4)	340 (2.0)	8154 (11.2)	762 (1.1)	178 (1.5)	186 (1.4)	1065 (1.2)	1448 (0.8)	4415 (1.0)	139 (1.2)	81 (0.7)	1594 (0.9)	17 950 (0.7)
Multiple sclerosis	2107 (8.0)	598 (3.5)	2012 (2.8)	2538 (3.8)	481 (4.1)	322 (2.5)	2433 (2.7)	16 473 (9.1)	17 508 (3.8)	273 (2.4)	49 (0.4)	3153 (1.8)	41 592 (1.6)
Myocardial infarction	388 (1.5)	169 (1.0)	4736 (6.5)	620 (0.9)	122 (1.0)	79 (0.6)	705 (0.8)	1583 (0.9)	3764 (0.8)	71 (0.6)	19 (0.2)	1094 (0.6)	11 810 (0.5)
Parkinson disease	330 (1.3)	153 (0.9)	275 (0.4)	278 (0.4)	115 (1.0)	44 (0.3)	1225 (1.3)	696 (0.4)	1680 (0.4)	31 (0.3)	10 (0.1)	258 (0.1)	3627 (0.1)
Peptic ulcer disease	2289 (8.7)	812 (4.7)	1650 (2.3)	1863 (2.8)	443 (3.8)	304 (2.4)	1806 (2.0)	5184 (2.9)	9162 (2.0)	222 (2.0)	43 (0.4)	2256 (1.3)	21 990 (0.9)
Peripheral artery disease	443 (1.7)	812 (4.7)	736 (1.0)	826 (1.2)	123 (1.0)	261 (2.0)	883 (1.0)	1617 (0.9)	5221 (1.1)	89 (0.8)	100 (0.9)	5395 (3.0)	17 045 (0.7)
Psoriasis	1862 (7.1)	232 (1.3)	2853 (3.9)	3238 (4.8)	730 (6.2)	7463 (58.0)	3334 (3.7)	6096 (3.4)	20 008 (4.3)	425 (3.7)	181 (1.6)	5127 (2.9)	44 700 (1.7)
Rheumatoid arthritis	608 (2.3)	835 (4.8)	1635 (2.2)	1045 (1.6)	193 (1.6)	96 (0.7)	1170 (1.3)	1839 (1.0)	6893 (1.5)	122 (1.1)	62 (0.6)	1394 (0.8)	27 521 (1.1)
Schizophrenia	4811 (18.3)	470 (2.7)	15 988 (22.0)	6992 (10.4)	1580 (13.4)	1213 (9.4)	7600 (8.4)	21 300 (11.8)	40 084 (8.7)	962 (8.5)	311 (2.8)	10 886 (6.1)	115 806 (4.5)
Stroke or transient ischemic attack	10 616 (40.5)	1987 (11.5)	45 797 (63.0)	36 624 (54.5)	6009 (50.9)	7703 (59.8)	53 434 (58.8)	88 878 (49.3)	268 479 (58.1)	7336 (64.7)	9806 (88.4)	114 051 (64.3)	193 3445 (75.2)

### Markers of Complexity by Physician Group

There was substantial variability across physician groups for all 9 of the complexity markers (Table 2; eTable 1 in the Supplement). Patients seen by nephrologists had the highest mean number of comorbidities (4.2; 95% CI, 4.2-4.3 vs [lowest] 1.1; 95% CI, 1.0-1.1), highest mean number of prescribed medications (14.2; 95% CI, 14.2-14.3 vs [lowest] 4.9; 95% CI, 4.9-4.9), highest rate of death (6.6%; 95% CI, 6.3%-6.9% vs [lowest] 0.1%; 95% CI, <0.1%-0.2%), and highest rate of placement in a long-term care facility (2.0%; 95% CI, 1.8%-2.2% vs [lowest] <0.1%; 95% CI, <0.1%-0.1%); patients seen by infectious disease specialists had the highest complexity as assessed by the other 5 markers: rate of a mental health condition (29%; 95% CI, 28%-29% vs [lowest] 14%; 95% CI, 14%-14%), mean number of physician types (5.5; 95% CI, 5.5-5.6 vs [lowest] 2.1; 95% CI, 2.1-2.1), mean number of physicians (13.0; 95% CI, 12.9-13.1 vs [lowest] 3.8; 95% CI, 3.8-3.8), mean days in hospital (15.0; 95% CI, 14.9-15.0 vs [lowest] 0.4; 95% CI, 0.4-0.4), and mean emergency department visits (2.6; 95% CI, 2.6-2.6 vs [lowest] 0.5; 95% CI, 0.5-0.5).

**Table 2.** Complexity Outcomes by Physician Type^a^

Physician Type	Comorbidities, Mean (95% CI), No.	Risk of Mental Health Condition (95% CI)	Prescribed Medications, Mean (95% CI), No.	Physician Types, Mean (95% CI), No.	Physicians, Mean (95% CI), No.	Days Spent in Hospital, Mean (95% CI), No.	Emergency Department Visits, Mean (95% CI), No.	Likelihood of Long-term Care Placement	Risk of Mortality
Nephrologist	4.2 (4.2-4.3)^b^	0.22 (0.22-0.23)^b^	14.2 (14.2-14.3)^b^	5.1 (5.1-5.1)^b^	11.0 (11.0-11.0)^b^	11.1 (11.0-11.1)^b^	1.7 (1.7-1.7)^b^	0.020 (0.018-0.022)^b^	0.066 (0.063-0.069)^b^
Infectious disease specialist	2.7 (2.7-2.8)	0.29 (0.28-0.29)^b^	12.0 (12.0-12.1)^b^	5.5 (5.5-5.6)^b^	13.0 (12.9-13.1)^b^	15.0 (14.9-15.0)^b^	2.6 (2.6-2.6)^b^	0.014 (0.012-0.016)^b^	0.043 (0.040-0.046)^b^
Neurologist	2.8 (2.8-2.8)	0.27 (0.26-0.27)^b^	9.6 (9.6-9.7)	4.2 (4.2-4.3)	7.9 (7.9-8.0)	5.6 (5.6-5.6)	1.3 (1.3-1.3)	0.011 (0.011-0.012)^b^	0.022 (0.021-0.023)
Respirologist	2.8 (2.8-2.8)	0.21 (0.21-0.22)	10.6 (10.6-10.6)	4.4 (4.3-4.4)	8.0 (8.0-8.0)	4.5 (4.4-4.5)	1.1 (1.1-1.1)	0.009 (0.008-0.010)	0.037 (0.036-0.039)
Hematologist	2.9 (2.8-2.9)^b^	0.20 (0.19-0.21)	10.3 (10.2-10.3)	5.0 (4.9-5.0)^b^	9.7 (9.7-9.8)^b^	8.2 (8.2-8.3)^b^	1.5 (1.5-1.6)^b^	0.010 (0.009-0.013)	0.050 (0.046-0.054)^b^
Rheumatologist	3.1 (3.0-3.1)^b^	0.19 (0.18-0.19)	10.7 (10.7-10.8)^b^	4.2 (4.1-4.2)	7.0 (7.0-7.0)	2.7 (2.7-2.7)	0.9 (0.9-0.9)	0.004 (0.003-0.005)	0.014 (0.012-0.016)
Gastroenterologist	2.3 (2.3-2.3)	0.21 (0.20-0.21)	8.6 (8.6-8.6)	4.1 (4.1-4.1)	7.5 (7.5-7.5)	4.1 (4.1-4.1)	1.0 (1.0-1.1)	0.006 (0.005-0.006)	0.023 (0.022-0.024)
Cardiologist	2.6 (2.6-2.6)	0.16 (0.16-0.16)	8.7 (8.7-8.7)	4.0 (4.0-4.0)	7.2 (7.2-7.2)	3.1 (3.1-3.1)	0.9 (0.9-0.9)	0.006 (0.006-0.007)	0.021 (0.020-0.021)
General internist	2.2 (2.2-2.2)	0.18 (0.18-0.18)	8.1 (8.0-8.1)	3.6 (3.6-3.6)	6.6 (6.6-6.6)	3.1 (3.1-3.1)	0.8 (0.8-0.8)	0.006 (0.006-0.007)	0.019 (0.018-0.019)
Endocrinologist	2.4 (2.4-2.4)	0.18 (0.17-0.19)	8.7 (8.7-8.8)	4.3 (4.2-4.3)	7.4 (7.4-7.5)	2.8 (2.8-2.9)	0.7 (0.7-0.7)	0.003 (0.002-0.004)	0.013 (0.011-0.015)
Allergist/ immunologist	1.1 (1.0-1.1)	0.15 (0.14-0.15)	6.4 (6.4-6.4)	3.5 (3.5-3.6)	5.8 (5.8-5.8)	0.4 (0.4-0.4)	0.6 (0.6-0.7)	0.000 (0.000-0.001)	0.001 (0.000-0.002)
Dermatologist	1.6 (1.6-1.6)	0.14 (0.14-0.14)	6.6 (6.6-6.6)	3.4 (3.4-3.4)	5.4 (5.4-5.4)	1.0 (0.9-1.0)	0.5 (0.5-0.5)	0.003 (0.003-0.003)	0.009 (0.009-0.009)
Family physician	1.3 (1.3-1.3)	0.14 (0.14-0.14)	4.9 (4.9-4.9)	2.1 (2.1-2.1)	3.8 (3.8-3.8)	1.0 (0.9-1.0)	0.6 (0.6-0.6)	0.003 (0.003-0.003)	0.008 (0.008-0.009)

^a^ Seven complexity markers were measured in the year prior to follow-up to avoid mortality bias: the number of comorbidities, the number of uniquely prescribed medications (defined by unique chemical entities as assessed by prescriptions filled), the presence of a mental health condition (defined by alcohol misuse, depression, or schizophrenia), the number of physician types seen by each patient, the total number of physicians involved in each patient’s care, the number of days spent in a hospital, and the number of emergency department visits. Two complexity markers were measured over the year of follow-up: the risk of new placement into long-term care and the risk of all-cause death.

^b^ The 3 highest unadjusted means or risks for each marker of complexity.

Between-group variability was most pronounced for mean number of days in the hospital and mean number of unique medications prescribed and least pronounced for long-term care placements and all-cause death (Table 2; eTable 1 in the Supplement). When complexity markers were expressed as the frequency of specific values rather than as means, these between-specialty differences became more apparent (eFigure 2 in the Supplement).

**Figure 1.**
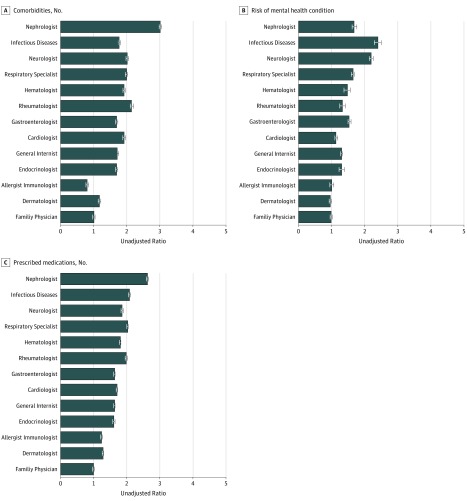
Relative Differences in 3 Complexity Markers, by Physician Type Error bars indicate 95% CIs. Relative differences in all 9 complexity markers (by physician type) can be found in eFigure 3 in the Supplement.

**Figure 2.**
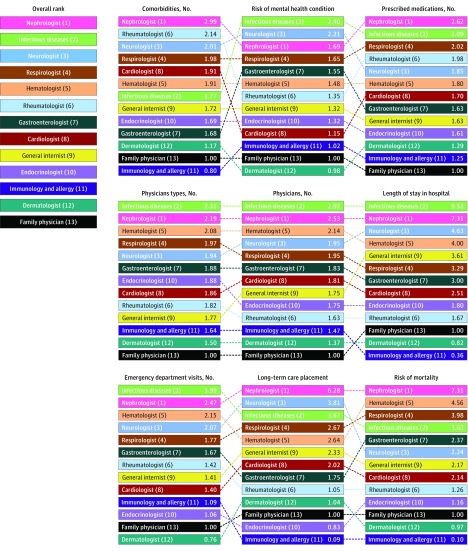
Complexity Rankings by Physician Type Using results from the regressions, the specialties were uniformly ranked for each marker of complexity. The ranks then were summed across complexities giving an overall complexity rank. Ties were broken using the highest frequency of the highest available rank between tied specialties.

There were clear trends in the average complexity of patients seen by physician type. Patients seen by infectious disease specialists, nephrologists, and neurologists were consistently more complex, and patients seen by endocrinologists, clinical allergists/immunologists, and dermatologists were consistently less complex (Table 2; eTable 1 in the Supplement). eFigure 3 in the Supplement expresses each of the complexity markers in relative terms (and Figure 1 expresses 3 of the complexity markers in relative terms), with each physician group compared with patients seen by family physicians. Overall ranking of patient complexity and individual ranking for each of the 9 complexity markers by physician group are shown in Figure 2. When types of physician were ranked according to patient complexity across all 9 markers, the order from most to least complex was nephrologist, infectious disease specialist, neurologist, respirologist, hematologist, rheumatologist, gastroenterologist, cardiologist, general internist, endocrinologist, allergist/immunologist, dermatologist, and family physician.

Results were consistent in sensitivity analyses that used each visit as the unit of analysis (giving more weight to patients who were seen multiple times [eTable 2 in the Supplement]), required more than 1 claim to define *being seen* by a particular specialty (eTables 3 and 4 in the Supplement), or repeated all analyses in a different time period (basing the cohort on Alberta residence to April 1, 2009, rather than April 1, 2014 [eTable 5 in the Supplement]). Considerable variability between specialties remained in all analyses, although there was some variation in the rankings. When the visit was used as the unit of analysis (or >1 claim was required to define being seen), the relative ranking of general internists and family physicians tended to increase, whereas the complexity of nephrology patients remained first overall, and the complexity of patients seen by infectious disease specialists, respiratory specialists, and neurologists were consistently ranked in the top 5. Repeating analyses using the 2009 cohort did not change any of the conclusions.

## Discussion

In keeping with our hypothesis, we found substantial differences in the average complexity of patients seen by different types of physician. Although no single specialty’s patients were most complex by all measures, patients seen by nephrologists, infectious disease specialists, and neurologists consistently tended to be more complex than others, whereas patients seen by other types of physician, such as clinical allergists, dermatologists, and family physicians, consistently tended to be less complex.

There is no agreed definition of patient complexity.^7^ Most available instruments, such as the Vector Model of Complexity^16^ or the Patient Centered Assessment Method,^2^ assess patients according to domains such as health, social factors, health literacy, and service coordination, each of which includes 2 or more subitems. Clinical experience and the available literature suggest that overall complexity includes not just medical issues but also social characteristics and is influenced by contextual factors, such as the structure and organization of the underlying health system. Given that it was based on administrative data, our analysis focused chiefly on medical aspects of complexity, although we included certain socioeconomic characteristics such as income, rural residence location, indigenous origin, and residence in a lower-income neighborhood, all of which were again more common in infectious diseases specialists and nephrologists. Our analysis would have been strengthened by availability of data to allow direct assessment of characteristics such as coordination of care rather than proxies. For example, a direct question such as “Are the services involved with this client well coordinated?” (as recommended by the Patient Centered Assessment Method^17^) would provide better insight as to the true complexity of a particular patient than simply counting the number of physician types involved in that patient’s care (as we did). However, while our approach has limitations, it should not have led to bias unless the proxies that we used are more or less accurate in some specialties than in others.

Although it seems widely accepted that the complexity of patients seen by different types of physician is highly variable, we did not identify other studies of this issue. Previous studies of complexity have tended to focus on the association between complexity (typically defined by number of morbidities alone) and clinical outcomes,^18,19^ or on the implications of complexity for health systems and health policy.^16,20,21^

Our primary analysis used the characteristics of the average patient seen by each specialty to assess complexity, which arguably best reflects the workload associated with a typical day of practice. However, this approach could be criticized on the grounds that physicians have little impact on the care of complex patients that they see only once. Using the visit (eTable 2 in the Supplement) as the unit of analysis (thus, giving greater weight to the characteristics of patients who are seen multiple times) partially addresses this limitation, as does retaining the patient as the unit of analysis but only including patients who saw each type of physician more than once (eTables 3 and 4 in the Supplement). We took both of these approaches in sensitivity analyses and found a similar overall ranking of specialties as compared with the primary analysis, with slightly larger differences between specialties. Repeating the analyses with an earlier cohort of patients demonstrated that results were robust over time.

The fact that the ranking was consistent regardless of the analytical approach taken should increase confidence in our findings. However, we believe that the relative rank of the different specialties we studied is less important than the finding that there are wide variations in complexity between specialties. The latter has potential implications for medical education and health policy. First, our findings suggest that skills in managing complex patients are more important for some specialties than for others, and that the skills required to care for complex patients should be considered when medical students choose a clinical specialty. Directors of residency programs in which complexity is especially common may consider the merits of including formal training on complexity, multimorbidity, and their implications. Second, there is no debate that patient complexity requires time (including the time required to communicate with the multiple other clinicians often involved in a patient’s care), expertise, and resources to optimize management. However, reimbursement of physicians and facilities in North America is most commonly based on fee-for-service compensation.^4^ In the fee-for-service payment structure, the type and duration of an encounter is the primary determinant of payment. The complexity of medical decision making is addressed by assessing the number of diagnoses and management options that are considered, the medical risks, and the amount of data to be reviewed. While easily ascertainable, these factors do not fully account for clinical complexity.^22,23,24^ Moreover, adjusting payments to encourage physicians or clinical programs to spend more time and resources caring for patients at highest risk of complications makes sense from a health care payer perspective. This is particularly important as health systems experiment with the use of bundled payment for hospital care for episodes of myocardial infarction or coronary artery bypass grafting, or for procedures like joint arthroplasty—where limited risk adjustment has been used to date.^25,26^ In view of our findings, policy makers should consider how funding for specialty-specific clinical programs and mechanisms for linking health care programs to social care initiatives could consider the complexity of patients more appropriately.^25,26,27^ This could be done by explicitly accounting for complexity when setting relative value units of evaluation and management codes^22^ as well as budgets for clinical programs, particularly in the context of bundled payments. Any such policy remedy would require careful consideration and rigorous evaluation in pilot testing before widespread adoption. Finally, we speculate that the observed differences in patient complexity may also contribute to differential burnout rates among medical specialties.^28^

Our study has several important strengths, including the use of population-based data from a geographically defined area served by a universal health care system; a relatively large sample size; use of validated algorithms for ascertaining the presence or absence of comorbidity and clinical outcomes; rigorous analytical methods; and consideration of a broad range of proxies for patient complexity.

### Limitations

Our study has limitations that should be considered when interpreting results. First, most of the authors of our study are nephrologists, and given the findings, there may be a perceived conflict of interest. We emphasize that the primary goal of this article was not to justify increased resources for kidney care programs specifically, but rather to propose a more nuanced consideration of how any health program is resourced in the face of increasing patient complexity. Second, like all studies using administrative data, some assumptions are required when assessing comorbidities, outcomes, and exposures. However, any misclassification should have been nondifferential and is unlikely to have affected the observed differences between physician types. In addition, it seems unlikely that nuances in billing practices or clinical practice patterns between different types of physician could completely explain the observed differences. Third, our data sources allowed us only to assess the presence or absence of comorbidity, rather than its severity. It is difficult to speculate how this might have affected our results, although it seems unlikely that better information on the severity of comorbidity would have affected our conclusions. Fourth, the presence of a comorbidity such as mental illness does not necessarily mean that physicians managed that comorbidity. Fifth, we studied people from a single Canadian province and our findings may not be generalizable to other health care settings. For example, in the United States, a lack of coordination between federal and state governments coupled with a complex mix of employer-sponsored and governmental health insurance could alter relative medical complexity by specialty. Sixth, we chose to include mortality and the likelihood of hospitalization as markers of complexity, although arguably these could be considered consequences of complexity instead. However, excluding these markers of complexity from our analysis would not have affected our main conclusions, especially if they were replaced with other candidate markers such as income, residence location, and indigenous origin. Seventh, and most important, we did not have data on other potentially important determinants of complexity such as adherence, opiate use, lack of fluency in one of Canada’s 2 official languages, health literacy, sensory impairment (eg, blindness or deafness), financial resources, or social networks.^29^

## Conclusions

We found substantial between-specialty differences in 9 different markers of patient complexity. These findings have implications for medical education and health policy.

## Notes

Supplement. **eTable 1. **Complexity Outcomes All Medical Subspecialists **eTable 2. **Complexity Outcomes All Medical Subspecialists—Sensitivity Analysis (Unit of Analysis Is Patient-Visit) **eTable 3.** Complexity Outcomes All Medical Subspecialists—Sensitivity Analysis (Seen at Least Twice) **eTable 4.** Complexity Outcomes All Medical Subspecialists—Sensitivity Analysis (Seen at Least Thrice) **eTable 5.** Complexity Outcomes All Medical Subspecialists—Sensitivity Analysis (Apr 2009-Mar 2010 Cohort) **eFigure 1.** Patient Flow Diagram **eFigure 2.** Distribution of Non-binary Complexity Markers, by Physician Type **eFigure 3.** Relative Differences in Complexity Markers, by Physician Type
