# Errors in Results, Table, and Figure

**DOI:** 10.1001/jamanetworkopen.2019.0147

**Published:** 2019-03-01

In the Original Investigation titled “Comparison of the Complexity of Patients Seen by Different Medical Subspecialists in a Universal Health Care System,”^1^ published November 30, 2018, there were errors in the Results section, Table 1, and Figure 2. In the Characteristics of Study Patients subsection of the Results section, the seventh sentence of the second paragraph should have read, “Patients of indigenous origin were most often seen by nephrologists, infectious disease specialists, and rheumatologists.” In Table 1, the following No. (%) values should have appeared in the social assistance row: neurologist, 6503 (9.0); respirologist, 4030 (6.0); gastroenterologist, 3957 (4.4); cardiologist, 6552 (3.6); and general internist, 20 232 (4.4). Also in Table 1, all morbidity values should have been shifted down by 1 row and the data in the last row should have been deleted. In Figure 2 in the physician types grouping, the means ratio for dermatologist should have appeared as 1.59. This article has been corrected.^1^ An explanation has been posted online.^2^
