# Supplementary material for: Comparison of the Complexity of Patients Seen by Different Medical Subspecialists in a Universal Health Care System
Source: JAMA Netw Open. 2018 Nov 30;1(7):e184852. doi: 10.1001/jamanetworkopen.2018.4852 (PMC6324421; doi:10.1001/jamanetworkopen.2018.4852)
Supplement: Supplement. — eTable 1. Complexity Outcomes All Medical Subspecialists eTable 2. Complexity Outcomes All Medical Subspecialists—Sensitivity Analysis (Unit of Analysis Is Patient-Visit) eTable 3. Complexity Outcomes All Medical Subspecialists—Sensitivity Analysis (Seen at Least Twice) eTable 4. Complexity Outcomes All Medical Subspecialists—Sensitivity Analysis (Seen at Least Thrice) eTable 5. Complexity Outcomes All Medical Subspecialists—Sensitivity Analysis (Apr 2009-Mar 2010 Cohort) eFigure 1. Patient Flow Diagram eFigure 2. Distribution of Non-binary Complexity Markers, by Physician Type eFigure 3. Relative Differences in Complexity Markers, by Physician Type [file jamanetwopen-1-e184852-s001.pdf]

Supplementary Online Content

Tonelli M, Wiebe N, Manns BJ, et al. Comparison of the complexity of patients seen by different medical subspecialists in a universal health care system. *JAMA Netw Open*. 2018;1(7):e184852. doi:10.1001/jamanetworkopen.2018.4852

eTable 1. Complexity Outcomes All Medical Subspecialists
eTable 2. Complexity Outcomes All Medical Subspecialists—Sensitivity Analysis (Unit of Analysis Is Patient-Visit)
eTable 3. Complexity Outcomes All Medical Subspecialists—Sensitivity Analysis (Seen at Least Twice)
eTable 4. Complexity Outcomes All Medical Subspecialists—Sensitivity Analysis (Seen at Least Thrice)
eTable 5. Complexity Outcomes All Medical Subspecialists—Sensitivity Analysis (Apr 2009-Mar 2010 Cohort)
eFigure 1. Patient Flow Diagram
eFigure 2. Distribution of Non-binary Complexity Markers, by Physician Type
eFigure 3. Relative Differences in Complexity Markers, by Physician Type

This supplementary material has been provided by the authors to give readers additional information about their work.

© 2018 Tonelli M et al. *JAMA Network Open.*

eTable 1. Complexity outcomes all medical subspecialists

Physician type	Mean number of comorbidities	Risk of mental health condition	Mean number of prescribed medications	Mean number of physician types	Mean number of physicians	Mean days spent in hospital	Mean number of emergency department visits	Likelihood of long-term care placement	Risk of mortality
Nephrologist	**2.99 (2.97,3.01)**	**1.69 (1.64,1.74)**	**2.62 (2.61,2.63)**	**2.19 (2.18,2.20)**	**2.53 (2.52,2.53)**	**7.31 (7.28,7.34)**	**2.47 (2.45,2.50)**	**6.28 (5.70,6.91)**	**7.31 (6.94,7.71)**
Infectious diseases	1.77 (1.76,1.79)	**2.40 (2.32,2.48)**	**2.09 (2.08,2.10)**	**2.31 (2.30,2.33)**	**2.92 (2.91,2.93)**	**9.52 (9.48,9.56)**	**3.99 (3.95,4.03)**	**3.67 (3.20,4.22)**	3.63 (3.35,3.94)
Neurologist	**2.01 (2.00,2.02)**	**2.21 (2.17,2.25)**	1.85 (1.84,1.85)	1.94 (1.93,1.94)	1.95 (1.95,1.96)	**4.63 (4.61,4.64)**	2.07 (2.06,2.08)	**3.81 (3.54,4.10)**	2.24 (2.13,2.36)
Respiratory	1.98 (1.97,1.99)	1.65 (1.62,1.68)	**2.02 (2.01,2.02)**	1.97 (1.96,1.97)	1.95 (1.94,1.95)	3.29 (3.28,3.31)	1.77 (1.75,1.78)	2.67 (2.45,2.92)	**3.98 (3.81,4.16)**
Hematologist	1.91 (1.89,1.93)	1.48 (1.41,1.55)	1.80 (1.79,1.81)	**2.08 (2.07,2.10)**	**2.14 (2.13,2.16)**	4.00 (3.97,4.02)	**2.15 (2.12,2.18)**	2.64 (2.19,3.18)	**4.56 (4.17,4.99)**
Rheumatologist	**2.14 (2.12,2.16)**	1.35 (1.29,1.41)	1.98 (1.97,1.99)	1.82 (1.81,1.84)	1.63 (1.62,1.64)	1.67 (1.65,1.69)	1.42 (1.39,1.44)	1.05 (0.79,1.39)	1.26 (1.08,1.47)
Gastroenterologist	1.68 (1.68,1.69)	1.55 (1.53,1.58)	1.63 (1.63,1.63)	1.88 (1.88,1.89)	1.83 (1.83,1.84)	3.00 (2.99,3.01)	1.67 (1.66,1.68)	1.75 (1.60,1.91)	2.37 (2.26,2.48)
Cardiologist	1.91 (1.90,1.92)	1.15 (1.13,1.16)	1.70 (1.70,1.70)	1.86 (1.85,1.86)	1.81 (1.81,1.82)	2.51 (2.51,2.52)	1.40 (1.39,1.40)	2.02 (1.90,2.15)	2.14 (2.07,2.22)
General internist	1.72 (1.72,1.72)	1.32 (1.31,1.33)	1.63 (1.63,1.63)	1.77 (1.77,1.78)	1.75 (1.75,1.75)	3.61 (3.60,3.61)	1.41 (1.40,1.41)	2.33 (2.23,2.42)	2.17 (2.12,2.22)
Endocrinologist	1.69 (1.67,1.71)	1.32 (1.25,1.38)	1.61 (1.60,1.62)	1.88 (1.86,1.90)	1.75 (1.74,1.76)	1.80 (1.78,1.82)	1.06 (1.04,1.09)	0.83 (0.59,1.18)	1.16 (0.98,1.37)
Immunol/allergy	0.80 (0.79,0.81)	1.02 (0.97,1.08)	1.25 (1.24,1.26)	1.64 (1.63,1.66)	1.47 (1.46,1.48)	0.36 (0.34,0.37)	1.09 (1.06,1.11)	0.09 (0.03,0.28)	0.10 (0.05,0.18)
Dermatologist	1.17 (1.17,1.18)	0.98 (0.97,1.00)	1.29 (1.29,1.29)	1.59 (1.58,1.59)	1.37 (1.37,1.38)	0.82 (0.81,0.82)	0.76 (0.75,0.76)	1.04 (0.95,1.12)	0.97 (0.92,1.01)
Family physician	1 (referent)	1 (referent)	1 (referent)	1 (referent)	1 (referent)	1 (referent)	1 (referent)	1 (referent)	1 (referent)

This table shows the means ratio or odds ratio as appropriate with 95% confidence intervals in brackets. Highest three unadjusted ratios are bolded. Physician type is ordered by rankings from the most complex overall to the least complex.

© 2018 Tonelli M et al. *JAMA Network Open.*

eTable 2. Complexity outcomes all medical subspecialists – sensitivity analysis (unit of analysis is patient-visit)

Physician type	Mean number of comorbidities	Risk of mental health condition	Mean number of prescribed medications	Mean number of physician types	Mean number of physicians	Mean days spent in hospital	Mean number of emergency department visits	Likelihood of long-term care placement	Risk of mortality
Nephrologist	**2.35 (2.35,2.35)**	1.13 (1.12,1.14)	**2.06 (2.06,2.06)**	**2.34 (2.34,2.35)**	**3.20 (3.20,3.20)**	**4.31 (4.30,4.31)**	**2.06 (2.05,2.07)**	**4.83 (4.73,4.94)**	**5.22 (5.16,5.28)**
Infectious diseases	**1.40 (1.39,1.41)**	**1.57 (1.54,1.61)**	**1.61 (1.61,1.62)**	**2.34 (2.30,2.31)**	**2.69 (2.68,2.69)**	**4.77 (4.76,4.78)**	**2.63 (2.61,2.64)**	**1.97 (1.83,2.11)**	1.75 (1.68,1.82)
Respiratory	**1.46 (1.46,1.47)**	**1.14 (1.13,1.15)**	1.55 (1.55,1.56)	1.88 (1.88,1.89)	1.79 (1.79,1.79)	2.66 (2.65,2.66)	1.40 (1.39,1.40)	1.74 (1.67,1.82)	**2.57 (2.52,2.62)**
Hematologist	1.33 (1.33,1.34)	0.88 (0.86,0.90)	**1.66 (1.66,1.67)**	**2.24 (2.24,2.25)**	**2.30 (2.29,2.30)**	**5.23 (5.22,5.24)**	**1.84 (1.83,1.86)**	1.45 (1.33,1.58)	**4.71 (4.57,4.84)**
Neurologist	1.37 (1.37,1.37)	**1.33 (1.32,1.35)**	1.24 (1.24,1.24)	1.74 (1.73,1.74)	1.57 (1.57,1.58)	2.70 (2.69,2.70)	1.33 (1.33,1.34)	**2.20 (2.11,2.28)**	1.06 (1.03,1.09)
General internist	1.37 (1.38,1.38)	1.03 (1.03,1.04)	1.30 (1.30,1.31)	1.67 (1.66,1.67)	1.65 (1.65,1.65)	2.58 (2.58,2.58)	1.29 (1.28,1.29)	1.86 (1.83,1.89)	1.73 (1.72,1.75)
Gastroenterologist	1.20 (1.20,1.20)	1.07 (1.06,1.09)	1.18 (1.18,1.18)	1.71 (1.71,1.71)	1.58 (1.58,1.58)	1.76 (1.75,1.75)	1.37 (1.36,1.37)	0.91 (0.86,0.96)	1.19 (1.16,1.22)
Cardiologist	1.39 (1.39,1.39)	0.68 (0.68,0.69)	1.28 (1.27,1.28)	1.68 (1.68,1.68)	1.54 (1.54,1.54)	1.37 (1.36,1.37)	1.08 (1.08,1.08)	0.95 (0.91,0.98)	1.10 (1.08,1.12)
Rheumatologist	1.39 (1.37,1.39)	0.74 (0.72,0.77)	1.31 (1.31,1.32)	1.58 (1.57,1.59)	1.17 (1.16,1.17)	0.60 (0.59,0.60)	0.82 (0.81,0.83)	0.48 (0.40,0.58)	0.41 (0.37,0.46)
Family physician	1 (referent)	1 (referent)	1 (referent)	1 (referent)	1 (referent)	1 (referent)	1 (referent)	1 (referent)	1 (referent)
Endocrinologist	1.06 (1.05,1.07)	0.70 (0.67,0.72)	1.02 (1.02,1.03)	1.65 (1.64,1.66)	1.35 (1.34,1.35)	0.98 (0.97,0.99)	0.66 (0.65,0.67)	0.40 (0.31,0.51)	0.41 (0.36,0.47)
Dermatologist	0.76 (0.76,0.75)	0.52 (0.52,0.52)	0.82 (0.82,0.82)	1.23 (1.23,1.24)	0.87 (0.87,0.87)	0.16 (0.16,0.17)	0.37 (0.37,0.37)	0.39 (0.37,0.41)	0.28 (0.27,0.29)
Immunol/allergy	0.40 (0.40,0.41)	0.48 (0.47,0.50)	0.66 (0.66,0.67)	1.26 (1.25,1.27)	0.85 (0.84,0.85)	0.05 (0.05,0.05)	0.40 (0.39,0.41)	0.01 (0.005,0.05)	0.01 (0.008,0.03)

This table shows the means ratio or odds ratio as appropriate with 95% confidence intervals in brackets. Highest three unadjusted ratios are bolded. Physician type is ordered by rankings from the most complex overall to the least complex. In this sensitivity analysis, the patient-visit was the unit of analysis.

© 2018 Tonelli M et al. *JAMA Network Open.*

eTable 3. Complexity outcomes all medical subspecialists – sensitivity analysis (seen at least twice)

Physician type	Mean number of comorbidities	Risk of mental health condition	Mean number of prescribed medications	Mean number of physician types	Mean number of physicians	Mean days spent in hospital	Mean number of emergency department visits	Likelihood of long-term care placement	Risk of mortality
Nephrologist	**2.63 (2.61,2.65)**	1.58 (1.52,1.65)	**2.37 (2.36,2.38)**	**2.19 (2.18,2.21)**	**2.48 (2.47,2.50)**	**6.13 (6.10,6.15)**	**2.42 (2.39,2.45)**	**5.85 (5.22,6.56)**	**7.19 (6.75,7.66)**
Infectious diseases	1.54 (1.52,1.55)	**2.23 (2.14,2.33)**	1.81 (1.80,1.82)	**2.23 (2.22,2.25)**	**2.67 (2.66,2.69)**	**6.71 (6.67,6.74)**	**3.42 (3.38,3.46)**	2.68 (2.24,3.20)	2.61 (2.35,2.90)
Respiratory	**1.99 (1.97,2.00)**	1.58 (1.53,1.63)	**2.03 (2.02,2.03)**	2.01 (2.00,2.02)	1.99 (1.98,2.00)	3.73 (3.71,3.75)	1.96 (1.94,1.98)	3.24 (2.88,3.63)	**5.38 (5.08,5.70)**
General internist	1.96 (1.95,1.97)	1.50 (1.48,1.51)	**1.86 (1.86,1.86)**	1.94 (1.93,1.94)	**2.09 (2.09,2.09)**	**6.13 (6.12,6.15)**	1.91 (1.90,1.92)	**3.28 (3.11,3.47)**	3.38 (3.27,3.48)
Neurologist	1.93 (1.91,1.94)	**2.18 (2.13,2.24)**	1.74 (1.73,1.75)	1.97 (1.95,1.98)	1.96 (1.95,1.96)	5.02 (5.00,5.04)	**2.17 (2.15,2.19)**	**4.51 (4.09,4.98)**	2.39 (2.22,2.57)
Hematologist	1.71 (1.69,1.74)	1.24 (1.16,1.33)	1.66 (1.65,1.67)	**2.03 (2.01,2.06)**	1.98 (1.96,1.99)	3.15 (3.12,3.17)	2.06 (2.02,2.10)	2.09 (1.60,2.72)	**4.88 (4.34,5.49)**
Cardiologist	**2.06 (2.05,2.07)**	1.09 (1.06,1.11)	1.82 (1.82,1.83)	1.95 (1.94,1.95)	2.01 (2.00,2.01)	2.84 (2.83,2.85)	1.71 (1.70,1.72)	2.07 (1.90,2.27)	2.43 (2.32,2.56)
Gastroenterologist	1.66 (1.65,1.67)	**1.64 (1.60,1.68)**	1.62 (1.61,1.62)	1.92 (1.91,1.93)	1.85 (1.85,1.86)	2.83 (2.82,2.84)	1.90 (1.88,1.91)	1.79 (1.59,2.02)	2.60 (2.44,2.76)
Rheumatologist	1.97 (1.94,2.00)	1.14 (1.06,1.22)	1.83 (1.82,1.85)	1.81 (1.79,1.83)	1.51 (1.50,1.53)	1.38 (1.36,1.40)	1.27 (1.24,1.31)	0.93 (0.62,1.40)	0.86 (0.67,1.10)
Endocrinologist	1.46 (1.43,1.49)	1.16 (1.05,1.27)	1.43 (1.41,1.44)	1.86 (1.83,1.89)	1.70 (1.68,1.72)	1.76 (1.73,1.80)	1.05 (1.01,1.09)	0.66 (0.34,1.28)	0.91 (0.66,1.26)
Family physician	1 (referent)	1 (referent)	1 (referent)	1 (referent)	1 (referent)	1 (referent)	1 (referent)	1 (referent)	1 (referent)
Dermatologist	1.11 (1.11,1.12)	0.83 (0.81,0.85)	1.21 (1.21,1.21)	1.54 (1.53,1.55)	1.25 (1.25,1.25)	0.63 (0.63,0.64)	0.66 (0.66,0.67)	1.03 (0.91,1.16)	0.95 (0.89,1.02)
Immunol/allergy	0.60 (0.57,0.62)	0.80 (0.70,0.90)	0.99 (0.97,1.01)	1.63 (1.59,1.66)	1.27 (1.25,1.29)	0.23 (0.21,0.24)	0.76 (0.72,0.81)	0.91 (0.89,0.93)	0.96 (0.95,0.97)

This table shows the means ratio or odds ratio as appropriate with 95% confidence intervals in brackets. Highest three unadjusted ratios are bolded. Physician type is ordered by rankings from the most complex overall to the least complex. In this sensitivity analysis, a patient was seen by a particular physician type if they were seen at least twice.

© 2018 Tonelli M et al. *JAMA Network Open.*

eTable 4. Complexity outcomes all medical subspecialists – sensitivity analysis (seen at least thrice)

Physician type	Mean number of comorbidities	Risk of mental health condition	Mean number of prescribed medications	Mean number of physician types	Mean number of physicians	Mean days spent in hospital	Mean number of emergency department visits	Likelihood of long-term care placement	Risk of mortality
Nephrologist	**2.38 (2.36,2.40)**	1.52 (1.45,1.60)	**2.17 (2.16,2.18)**	**2.22 (2.20,2.24)**	**2.58 (2.56,2.59)**	**5.24 (5.21,5.26)**	**2.37 (2.34,2.41)**	**5.40 (4.72,6.18)**	**6.87 (6.38,7.41)**
General internist	**1.98 (1.98,1.99)**	1.54 (1.51,1.56)	**1.87 (1.87,1.87)**	1.99 (1.99,2.00)	**2.20 (2.20,2.21)**	**7.25 (7.23,7.27)**	2.13 (2.12,2.14)	**3.75 (3.52,4.00)**	4.18 (4.03,4.33)
Infectious diseases	1.51 (1.49,1.53)	**2.20 (2.08,2.33)**	1.73 (1.72,1.75)	**2.25 (2.22,2.27)**	**2.68 (2.66,2.70)**	**6.00 (5.97,6.04)**	**3.13 (3.09,3.18)**	2.70 (2.19,3.34)	2.33 (2.05,2.66)
Respiratory	**1.89 (1.87,1.91)**	1.51 (1.45,1.58)	**1.94 (1.93,1.95)**	2.02 (2.00,2.04)	2.00 (1.99,2.01)	3.58 (3.56,3.60)	2.00 (1.97,2.02)	3.15 (2.71,3.66)	**6.12 (5.70,6.57)**
Neurologist	1.83 (1.82,1.85)	**2.10 (2.02,2.18)**	1.66 (1.65,1.67)	1.99 (1.97,2.00)	1.99 (1.98,2.00)	5.35 (5.32,5.37)	**2.20 (2.17,2.22)**	**4.87 (4.30,5.52)**	2.40 (2.18,2.64)
Hematologist	1.57 (1.53,1.60)	1.02 (0.93,1.12)	1.61 (1.59,1.63)	**2.04 (2.00,2.07)**	1.97 (1.95,1.99)	3.16 (3.13,3.20)	2.08 (2.03,2.13)	1.67 (1.18,2.36)	**5.24 (4.55,6.04)**
Gastroenterologist	1.62 (1.60,1.63)	**1.69 (1.64,1.76)**	1.57 (1.56,1.58)	1.92 (1.91,1.93)	1.91 (1.90,1.91)	2.85 (2.83,2.87)	2.08 (2.06,2.11)	1.81 (1.53,2.13)	2.75 (2.53,2.98)
Cardiologist	1.96 (1.95,1.97)	0.95 (0.92,0.98)	1.75 (1.75,1.76)	1.93 (1.93,1.94)	2.00 (1.99,2.01)	2.68 (2.67,2.69)	1.72 (1.70,1.73)	1.86 (1.66,2.09)	2.25 (2.11,2.39)
Rheumatologist	1.79 (1.75,1.83)	1.11 (1.00,1.23)	1.75 (1.73,1.77)	1.79 (1.76,1.83)	1.48 (1.46,1.50)	1.32 (1.30,1.35)	1.26 (1.21,1.31)	0.97 (0.56,1.68)	0.67 (0.46,0.97)
Endocrinologist	1.32 (1.27,1.37)	0.87 (0.75,1.02)	1.31 (1.29,1.34)	1.91 (1.87,1.96)	1.82 (1.79,1.85)	1.96 (1.92,2.01)	1.04 (0.98,1.10)	0.78 (0.32,1.89)	0.90 (0.56,1.43)
Family physician	1 (referent)	1 (referent)	1 (referent)	1 (referent)	1 (referent)	1 (referent)	1 (referent)	1 (referent)	1 (referent)
Dermatologist	1.01 (1.00,1.01)	0.72 (0.70,0.74)	1.11 (1.10,1.11)	1.46 (1.45,1.47)	1.14 (1.14,1.15)	0.46 (0.45,0.46)	0.59 (0.58,0.60)	0.83 (0.71,0.99)	0.80 (0.73,0.89)
Immunol/allergy	0.45 (0.42,0.48)	0.59 (0.49,0.71)	0.78 (0.76,0.80)	1.48 (1.43,1.53)	1.07 (1.04,1.10)	0.15 (0.13,0.17)	0.46 (0.42,0.51)	0.64 (0.63,0.66)	0.66 (0.65,0.67)

This table shows the means ratio or odds ratio as appropriate with 95% confidence intervals in brackets. Highest three unadjusted ratios are bolded. Physician type is ordered by rankings from the most complex overall to the least complex. In this sensitivity analysis, a patient was seen by a particular physician type if they were seen at least thrice.

© 2018 Tonelli M et al. *JAMA Network Open.*

eTable 5. Complexity outcomes all medical subspecialists – sensitivity analysis (Apr 2009-Mar 2010 cohort)

Physician type	Mean number of comorbidities	Risk of mental health condition	Mean number of prescribed medications	Mean number of physician types	Mean number of physicians	Mean days spent in hospital	Mean number of emergency department visits	Likelihood of long-term care placement	Risk of mortality
Nephrologist	**3.05 (3.03,3.07)**	1.58 (1.53,1.64)	**2.79 (2.77,2.80)**	**2.19 (2.17,2.20)**	**2.44 (2.43,2.45)**	**6.63 (6.60,6.67)**	**2.45 (2.42,2.47)**	**4.34 (4.10,4.58)**	**8.04 (7.77,8.31)**
Infectious diseases	1.73 (1.71,1.75)	**2.42 (2.32,2.53)**	**2.10 (2.09,2.11)**	**2.35 (2.33,2.37)**	**2.84 (2.83,2.86)**	**8.78 (8.73,8.82)**	**3.81 (3.76,3.86)**	2.05 (1.87,2.25)	3.29 (3.12,3.48)
Respiratory	**2.13 (2.12,2.14)**	**1.77 (1.73,1.82)**	**2.21 (2.20,2.22)**	2.05 (2.04,2.06)	2.04 (2.03,2.05)	3.98 (3.96,3.99)	1.91 (1.90,1.93)	**2.45 (2.33,2.57)**	**4.14 (4.02,4.26)**
Neurologist	**2.02 (2.01,2.03)**	**2.28 (2.24,2.33)**	1.90 (1.89,1.90)	1.93 (1.92,1.94)	1.91 (1.91,1.92)	4.44 (4.42,4.46)	**2.10 (2.08,2.11)**	**3.40 (3.28,3.52)**	2.41 (2.35,2.48)
Hematologist	1.89 (1.86,1.93)	1.62 (1.49,1.76)	1.84 (1.82,1.86)	**2.13 (2.09,2.16)**	**2.17 (2.15,2.19)**	**4.83 (4.78,4.87)**	1.92 (1.87,1.97)	1.82 (1.53,2.15)	**5.10 (4.68,5.55)**
General internist	1.84 (1.84,1.85)	1.34 (1.33,1.35)	1.77 (1.77,1.78)	1.84 (1.84,1.84)	1.77 (1.77,1.78)	4.24 (4.23,4.25)	1.51 (1.50,1.52)	2.25 (2.21,2.29)	2.30 (2.28,2.33)
Gastroenterologist	1.72 (1.72,1.73)	1.61 (1.58,1.65)	1.71 (1.70,1.71)	1.92 (1.91,1.92)	1.87 (1.86,1.87)	3.75 (3.73,3.76)	1.69 (1.68,1.70)	1.63 (1.56,1.70)	2.15 (2.10,2.21)
Rheumatologist	1.99 (1.97,2.01)	1.44 (1.39,1.50)	2.02 (2.01,2.03)	1.84 (1.82,1.85)	1.66 (1.65,1.67)	1.51 (1.50,1.52)	1.46 (1.43,1.48)	1.48 (1.36,1.61)	1.50 (1.42,1.59)
Cardiologist	1.81 (1.80,1.81)	1.12 (1.10,1.13)	1.61 (1.61,1.61)	1.82 (1.82,1.83)	1.75 (1.75,1.76)	2.41 (2.40,2.42)	1.19 (1.19,1.20)	1.92 (1.87,1.97)	2.16 (2.12,2.20)
Endocrinologist	1.63 (1.61,1.65)	1.35 (1.28,1.43)	1.64 (1.63,1.65)	1.92 (1.90,1.93)	1.79 (1.78,1.81)	1.79 (1.77,1.81)	1.08 (1.05,1.11)	1.25 (1.10,1.42)	1.21 (1.12,1.32)
Immunol/allergy	0.85 (0.83,0.88)	1.19 (1.10,1.28)	1.35 (1.34,1.37)	1.67 (1.64,1.69)	1.54 (1.52,1.56)	0.39 (0.37,0.41)	1.19 (1.15,1.23)	0.29 (0.20,0.41)	0.19 (0.14,0.24)
Dermatologist	1.16 (1.15,1.16)	1.02 (1.01,1.04)	1.29 (1.29,1.29)	1.61 (1.60,1.61)	1.38 (1.37,1.38)	0.83 (0.82,0.83)	0.69 (0.68,0.70)	1.19 (1.15,1.23)	1.12 (1.10,1.15)
Family physician	1 (referent)	1 (referent)	1 (referent)	1 (referent)	1 (referent)	1 (referent)	1 (referent)	1 (referent)	1 (referent)

This table shows the means ratio or odds ratio as appropriate with 95% confidence intervals in brackets. Highest three unadjusted ratios are bolded. Physician type is ordered by rankings from the most complex overall to the least complex.

© 2018 Tonelli M et al. *JAMA Network Open.*

eFigure 1. Patient flow diagram

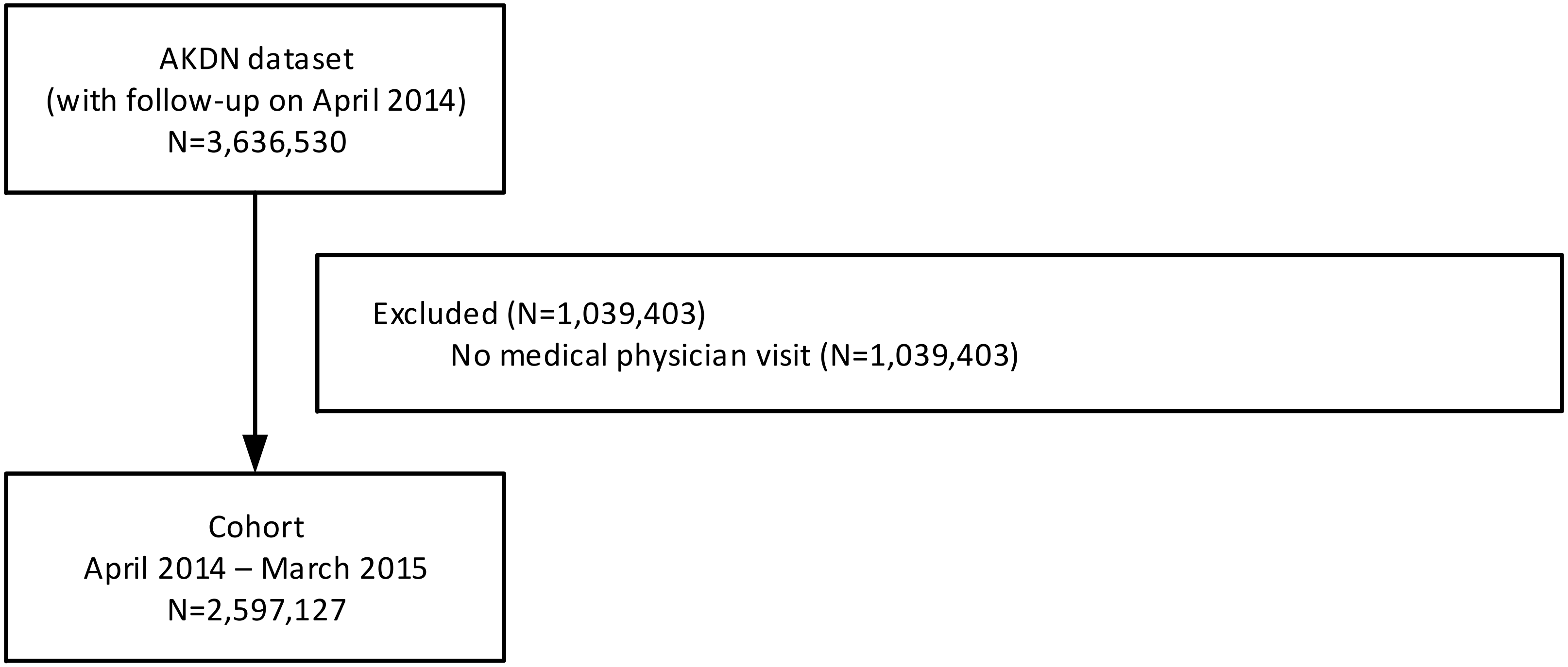

AKDN Alberta Kidney Disease Network

© 2018 Tonelli M et al. *JAMA Network Open.*

eFigure 2. Distribution of non-binary complexity markers, by physician type

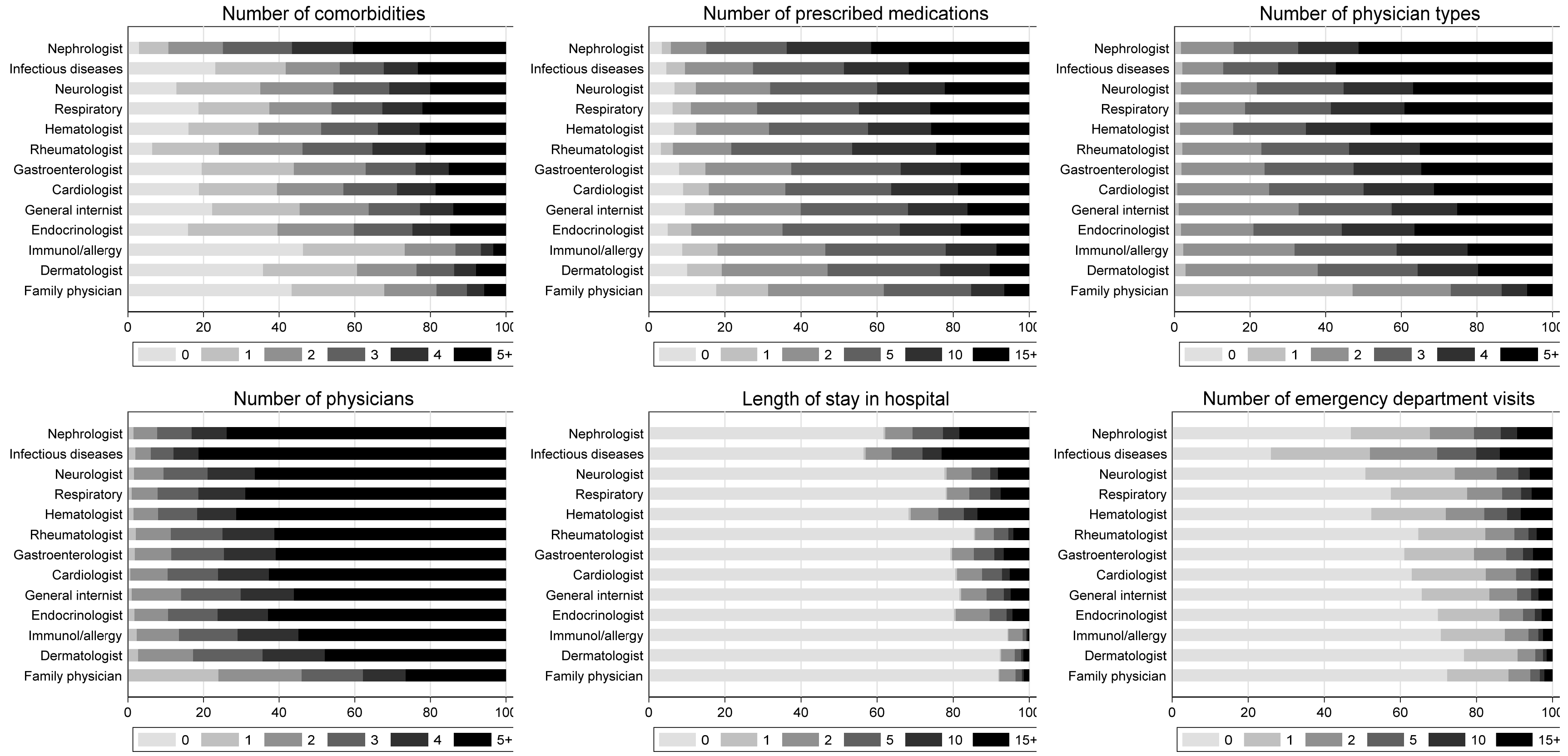

From left to right in the top row, the figure shows the frequencies for specific numbers of comorbidities, numbers of prescriptions, numbers of physician types. From left to right in the bottom row, the figure shows the frequencies for the specific numbers of physicians, numbers of days spent in hospital, and numbers of emergency visits. Physician types are ordered on the y-axis according to the overall complexity ranking presented in Figure 2.

© 2018 Tonelli M et al. *JAMA Network Open.*

eFigure 3. Relative differences in complexity markers, by physician type

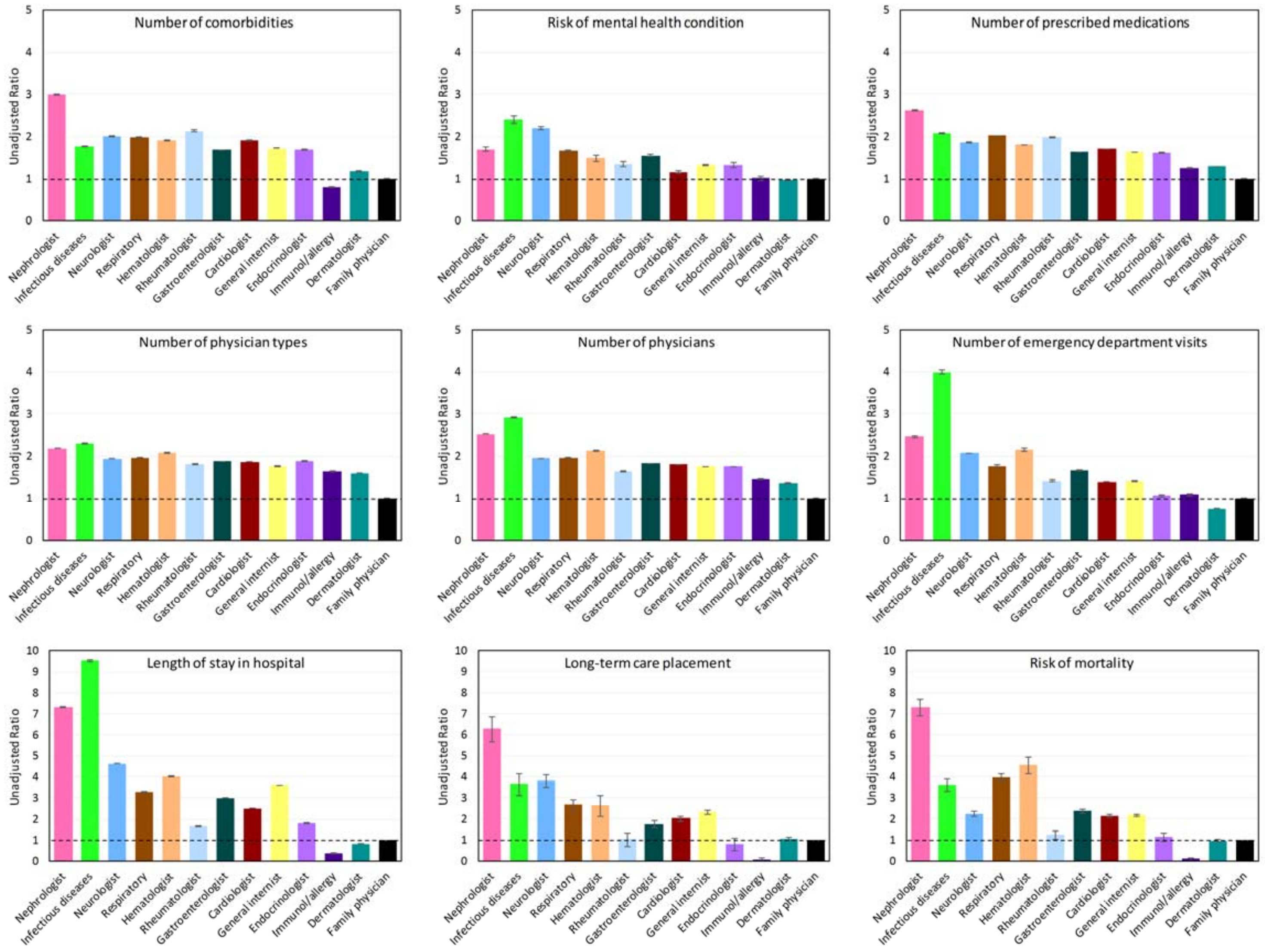

© 2018 Tonelli M et al. *JAMA Network Open.*
